# Designing a Gamified mHealth App for HIV Prevention and Comorbidities Among Malaysian Young Men Who Have Sex With Men: An Interdisciplinary Expert Panel Study

**DOI:** 10.2196/80334

**Published:** 2026-09-08

**Authors:** Xin Zhou, Ersin Dincelli, Frederick L Altice, Roman Shrestha, Lisa Hightow-Weidman, Iskandar Azwa, Rong Xiang Ng, Kamal Gautam, Md Safaet Hossain Sujan, Kiran Paudel, Jeffrey A Wickersham

**Affiliations:** 1 AIDS Program, School of Medicine, Yale University, 135 College St, New Haven, CT, 06510, United States, 1 2037374158; 2 Department of Information Systems, Business School, University of Colorado Denver, Denver, CO, United States; 3 Department of Allied Health Sciences, University of Connecticut, Storrs, CT, United States; 4 The Institute on Digital Health and Innovation, College of Nursing, Florida State University, Tallahassee, FL, United States; 5 Department of Infectious Diseases, University of Malaya, Kuala Lumpur, Malaysia; 6 Department of Medicine, Universiti Malaya, Kuala Lumpur, Malaysia

**Keywords:** mHealth, gamification, HIV prevention, young men who have sex with men, YMSM, stigma, privacy, integrated behavioral model, expert panel, mobile health

## Abstract

**Background:**

New HIV cases among Malaysian men who have sex with men continue to rise, with young men who have sex with men (YMSM) accounting for 44% of new infections and experiencing high rates of comorbidities. Mobile health (mHealth) apps offer a promising approach to addressing these challenges by providing discreet access to health information, screening tools, and linkage to services. Given the near-universal smartphone ownership among Malaysian YMSM and high levels of mobile gaming engagement, gamified mHealth apps may be particularly effective in sustaining engagement and promoting HIV prevention behaviors and comorbidity management. However, realizing their full potential requires identifying the features and design principles that are most important to Malaysian YMSM and that can support sustained engagement and improve health outcomes in this vulnerable population.

**Objective:**

This study used an interdisciplinary expert panel approach to identify the key features, design principles, and gamification elements to be incorporated into MY-Hero, a gamified mHealth app designed to support HIV prevention, comorbidity management, and overall well-being among Malaysian YMSM.

**Methods:**

Guided by the integrated behavioral model (IBM), we conducted an interdisciplinary expert panel comprising experts in health, technology, and design sciences, as well as health care professionals with expertise in the Malaysian YMSM population, along with local lesbian, gay, bisexual, transgender, queer, and others (LGBTQ+) community leaders. Through a structured series of discussions and thematic analysis, we identified culturally appropriate design principles and key features for MY-Hero that align with the health needs and sociocultural context of Malaysian YMSM.

**Results:**

Three expert panel sessions were conducted with 9 experts. Several key themes emerged: (1) the importance of establishing clinical affiliation and facilitating linkage to HIV testing, pre-exposure prophylaxis (PrEP), and related services, including harm reduction services; (2) the need to enhance user interface (UI) and user experience (UX) by optimizing usability, interactivity, and engagement to maintain user interest; (3) the incorporation of customizable health content to tailor interventions based on individual characteristics and preferences; (4) the use of gamification mechanisms, such as reward systems and progression tracking to promote adoption and sustained engagement with health services; and (5) the importance of privacy and data security as critical design considerations for ensuring user safety, confidentiality, and trust.

**Conclusions:**

The findings highlight the importance of user-centered design, contextualized health content, and gamification mechanisms in mHealth tools for Malaysian YMSM. These insights provide practical guidance for developing culturally appropriate gamified mHealth interventions for vulnerable populations by informing strategies to enhance user engagement, reduce HIV prevention fatigue, and support the well-being of YMSM in stigmatized contexts.

## Introduction

### Malaysian Young Men Who Have Sex With Men Face Higher Risk for HIV Infection and Related Comorbidities

Despite global declines in HIV incidence and mortality, this progress remains uneven across many geographic regions and key affected populations, such as men who have sex with men (MSM), especially in Malaysia, where HIV incidence among MSM continues to increase [1-3]. Recent estimates indicate that HIV prevalence among MSM has reached an all-time high of 21.6% [4], with especially alarming trends in Kuala Lumpur, where HIV prevalence remains high and appears to be increasing [3]. Young men who have sex with men (YMSM; younger than 29 years old) in Malaysia, in particular, account for 44% of new HIV infections in Malaysia [5], driven by high rates of condomless sex, low uptake of HIV testing, and limited access to pre-exposure prophylaxis (PrEP) [6-9]. In addition to heightened risk for HIV infection, Malaysian YMSM also experience high levels of comorbid medical and psychiatric conditions, including high rates of tobacco use [10], alcohol consumption, and methamphetamine use [6,11,12], mental disorders [13,14], and sexually transmitted infections (STIs) [15-18]. Furthermore, their misperceptions of invulnerability and related impulsive acts, and lack of accurate knowledge on the association between these comorbidities and their HIV risk, are associated with lower perceived well-being [19,20]. These overlapping comorbid conditions not only contribute to elevated risk-taking [21,22], but also undermine engagement in HIV prevention services, such as HIV testing and PrEP. A more holistic approach that addresses these intersecting comorbid conditions may significantly reduce HIV risk and improve overall health outcomes among Malaysian YMSM.

### Potential of Gamified mHealth Apps for HIV Prevention in Malaysian YMSM

In Malaysia, where same-sex sexual activity is criminalized under both secular and Shariah law, YMSM face significant legal and social barriers to accessing HIV prevention and related care [23]. These punitive legal frameworks contribute to pervasive stigma and discrimination, often reinforced in health care and clinical settings, which can deter YMSM from seeking in-person services [2]. Mobile apps offer a promising digital solution by providing safe and user-controlled access to information, screening tools, and linkage to services. By bypassing face-to-face interactions with potentially stigmatizing providers, mobile apps can reduce opportunities for enacted stigma, thus creating safer and more affirming pathways to care. In this context, digital platforms may not only improve engagement in HIV prevention and mental health services but also help restore autonomy, confidentiality, and dignity among YMSM who would otherwise avoid traditional care settings due to fear of exposure, judgment, or legal repercussions [24-26]. As such, mobile apps represent a scalable and context-sensitive approach to reducing stigma-related barriers and supporting the health and well-being of this highly marginalized population. Moreover, these apps have the potential to serve as a “one-stop-shop” model to address these complex and overlapping conditions faced by Malaysian YMSM. Fortunately, smartphone ownership is nearly universal in this population (97%) [27,28] with the majority (63%) frequently using apps for health information seeking [28], highlighting the feasibility and acceptability of mHealth apps for HIV prevention and care in this vulnerable population.

Many existing HIV prevention apps, however, endure low user interest and poor retention, especially among YMSM. Studies of mobile health interventions indicate that less than 20%‐40% of users remain active after a few months, and many lose engagement shortly after initial use (eg, only approximately 19% completed activities in the “Stick To It” HIV/STI screening app) [29]. HIV prevention fatigue, resulting from repetitive exposure to traditional HIV prevention interventions, such as persistent recommendations for condom use, can also undermine gains in this group due to boredom and diminishing interest in existing HIV prevention methods, which can lead to increased condomless sex [30-32]. Gamification, the integration of game design elements such as rewards, social incentives (eg, competition and collaboration), goal-setting, storytelling, and elements of chance and surprise into nongame contexts [33-35], can address the issues of low engagement and HIV prevention fatigue. More broadly, gamification strategies have been associated with higher long-term engagement in digital health tools, often boosting motivation and adherence to healthier behaviors [36,37], and better health outcomes [34] in areas such as depression, diabetes, cancer, nutrition, and sexual health [38].

In the context of HIV, gamification has shown promise in maintaining user interest and improving retention in HIV-related mHealth tools [39-43]. A recent meta-analysis of gamified interventions among MSM found significant short-term reductions in condomless anal sex and improved PrEP adherence [44]. Furthermore, qualitative evaluations of gamified platforms reveal that point-based rewards and social connectivity increased acceptability and ongoing use among MSM users [45]. By leveraging these game mechanics, HIV prevention apps can overcome attrition, sustain behavior change, and thereby enhance their public health impact. Gamification can also be particularly useful in addressing comorbidities among YMSM, including substance use, smoking, depression, and stigma, which require sustained efforts in self-regulation and adherence. Transferring these efforts into a fun and engaging digital environment may influence users’ health attitudes, self-efficacy in behavioral change, and intrinsic motivation toward health-promoting behaviors, all of which are associated with greater adherence to health-promoting activities [46,47]. Expanding app functions to track additional health domains (eg, smoking, physical activity, mood, and alcohol and drug use) may also diversify content, reduce HIV prevention fatigue, and position the app as a comprehensive “one-stop” platform that integrates HIV and non-HIV health needs while helping to destigmatize prevention [40].

Despite evidence that gamified apps enhance adherence to both HIV treatment [39-41] and PrEP [42] and promote HIV testing [43] in the US context, gamified HIV prevention apps have not yet been adapted for Malaysian YMSM. This presents a gap given the digital gaming culture among Malaysian YMSM, with 61.5% reporting gaming engagement [48] and 80% primarily using smartphones for digital gaming [49], suggesting high feasibility and scalability of gamified HIV prevention apps. In a context marked by rising HIV incidence, persistent structural and stigma-related barriers to prevention and care, limited culturally tailored programs, and growing fatigue toward traditional HIV prevention methods, culturally adapted gamified strategies offer an innovative and engaging approach to sustain prevention behaviors and reduce HIV incidence in this key affected population.

### Using the Integrated Behavioral Model to Design a Gamified mHealth App

The alpha version of the My-Hero was adapted from an existing mHealth platform, HealthMPowerment (HMP), which was developed based on the integrated behavioral model (IBM) [50,51]. The IBM extends the theory of planned behavior (TPB) by including 5 additional components known to impact HIV prevention behaviors, including affective motivations, behavioral skills and capacity, perceived and actual environmental constraints, prior habits, and salience of these behaviors. This model recognizes that decision-making is often affectively, rather than analytically, motivated and acknowledges that YMSM’s lives may be impacted by intersectional stigma, psychological distress, and substance use, which hinder adoption and maintenance of HIV prevention behaviors. In the context of sexual health, the IBM has been used to guide mHealth strategies in HIV and STI testing and PrEP uptake. Specifically, educational content, motivational messaging, and app features were designed to incorporate the IBM components, aiming to increase motivational beliefs, perceived norms and perceived behavioral control, attitudes toward testing, and self-efficacy, which then led to improvements in routine HIV and STI testing and PrEP uptake in both clinical trials and observational studies [52,53]. Building on this foundation, the IBM guided the design and development of the key features of HMP, including gamification elements, to ensure its breadth and interactivity by allowing MSM to (1) learn credible HIV prevention content through HMP’s curated content library; (2) customize HMP’s content by discussing relevant topics with clinicians and through conversations with peers; (3) engage in gamified activities that elicit reflection on environments, habits, behaviors, and motivations as a means to identify new risk reduction strategies; and (4) practice skills through interactive activities and games to reinforce self-efficacy and behavioral skills. Accordingly, MY-Hero includes multiple features that address these important domains, including information (education modules), motivation (gamification incentives and normative encouragement), and behavioral skills (through interactive tools and practice challenges). Multimedia Appendix 1A presents the key features and functionalities of HMP. Multimedia Appendix 1B presents their alignment with the IBM. Building on this foundation, this study aims to adapt HMP to the Malaysian context by identifying app features, design principles, and gamification elements that are most relevant and culturally appropriate for Malaysian YMSM. To achieve this, we employed an interdisciplinary expert panel approach to systematically elicit and refine recommendations that would enhance the app’s cultural relevance, feasibility, and potential public health impact.

### An Interdisciplinary Expert Panel Approach in mHealth Research

mHealth research is interdisciplinary by nature. The successful design and implementation of mHealth tools require expertise spanning applied computing, medicine, health care information systems, human-computer interaction, collaborative technologies, and social sciences [54]. Integrating knowledge from these domains is essential to understand how mHealth tools can be effectively used for disease prevention [55]. As technology continues to evolve rapidly, an interdisciplinary approach to mHealth tool development is particularly important to keep pace with technological advancements and address complex health care challenges [56,57]. Collaborating with interdisciplinary expert groups from different fields brings broader perspectives and enhances the quality, relevance, and impact of mHealth research [56,58], ensuring that they are not only effective in improving targeted health outcomes but also user-friendly, culturally appropriate, and responsive to the diverse needs of the target population, particularly in stigmatized cultural contexts such as Malaysia, where stigma negatively impacts health behaviors and overall well-being.

In the context of health, the expert panel approach uses structured discussions that integrate domain-specific expertise to guide the design, refinement, and implementation of health interventions, which is particularly useful for complex and interdisciplinary studies.

Expert panels are especially valuable when empirical evidence is limited or when multiple disciplinary perspectives are needed to inform intervention development and decision-making [59,60]. Expert panels have been used in mHealth studies, including physical activity [61], breastfeeding [62], obesity management [63], anemia and epilepsy prevention [64,65]. Unlike traditional qualitative methods such as focus groups or interviews, an interdisciplinary expert panel convenes experts from multiple fields who possess extensive experience and contextual knowledge of the relevant sociocultural and technological landscape. This approach enables the generation of practice-oriented insights that extend beyond theoretical frameworks, thereby helping bridge the gap between academic knowledge and real-world practice [66]. Expert panels are particularly valuable when a study involves hard-to-reach populations, such as YMSM in stigmatized contexts, where direct engagement may be more sensitive, complex, or limited [67]. Even when the target population is accessible, consulting experts at an early stage helps ensure that the app is appropriately designed before end-user involvement. This is especially important because poorly designed apps may not only fail to meet users’ needs but may also inadvertently cause harm or discourage user engagement among vulnerable populations [68]. Additionally, in the context of mHealth for HIV prevention among MSM, the interdisciplinary expert panel approach has not been widely used. This presents a valuable opportunity to align app design with end-user needs while prioritizing usability and accessibility, thus strengthening the development, relevance, and effectiveness of such interventions.

Given the potential of gamified mHealth tools to address HIV prevention and comorbidity management needs among Malaysian YMSM, this study aimed to use an interdisciplinary expert panel approach to identify key app features, design principles, and gamification elements of the MY-Hero app with the goal of making it culturally appropriate and tailored to HIV prevention and comorbidity management needs of this population.

## Methods

### Design

We collected data using an interdisciplinary expert panel approach that incorporated structured panel discussions to gather and synthesize expert insights to (1) identify essential app features for HIV prevention and (2) explore potential improvements (eg, design principles) to maximize the app’s impact among Malaysian MSM. The expert panel was composed of researchers with extensive experience in health sciences, technology, and design sciences, as well as health care professionals and local LGBTQ+ community leaders with expertise in the sociocultural factors that are unique to the Malaysian context and the Malaysian YMSM population.

### Participants

Experts were recruited using a convenience sampling strategy to ensure representation across 3 key domains relevant to the development of the MY-Hero app. Convenience sampling is commonly used in studies employing expert panels, often through established professional networks or other readily accessible experts [69-72]. In this study, experts were identified through the research team’s professional networks and referral recommendations. We assembled three expert panels representing (1) health sciences, including infectious diseases, HIV, and STIs; (2) technology and design sciences, including eHealth, mHealth development, and UX design; and (3) Malaysian lesbian, gay, bisexual, transgender, queer, and other (LGBTQ+) community leadership and advocacy.

Experts were selected based on their demonstrated expertise in their respective fields, including strong scientific credentials, a high publication record, or substantial professional experience. For the technology and design sciences panel, inclusion criteria required each expert to have either a doctoral degree and at least 1500 citations or an h-index greater than 10, or more than 10 years of experience designing gamified apps, or more than 10 years of experience designing and implementing mHealth tools for care delivery. For the health sciences and Malaysian context panels, inclusion criteria required at least 10 years of clinical experience in HIV prevention and care or at least 10 years of community service experience in the Malaysian context.

We identified 12 experts (5 leaders from the LGBTQ+ community, 4 from technology and design sciences, and 3 from health sciences) and invited each of them via email to participate in the study. Of these 12 experts, 9 accepted the invitation, with 3 experts participating in each panel. Table 1 presents the characteristics of the experts in each panel. To minimize potential bias, all invited experts were asked to disclose any potential conflicts of interest related to the development or evaluation of digital health technologies or HIV prevention interventions. None reported financial or professional conflicts of interest that would have influenced their contributions to the panel discussions. In addition, the experts were not involved in the direct development of the MY-Hero app and were invited specifically to provide independent perspectives on its development.

**Table 1.** Characteristics of the experts (N=9).

Characteristics	Technology sciences, n (%)	Health sciences, n (%)	Malaysian LGBTQ+^a^ , n (%)
Sex			
Male	3 (33.3)	2 (22.2)	3 (33.3)
Female	0 (0)	1 (11.1)	0 (0)
Expertise			
Addiction	0 (0)	1 (11.1)	0 (0)
Infectious diseases	0 (0)	1 (11.1)	0 (0)
Mental health	0 (0)	1 (11.1)	0 (0)
Local community for organizing events	0 (0)	0 (0)	2 (22.2)
Malaysian MSM^b^ advocates	0 (0)	0 (0)	1 (11.1)
eHealth and mHealth^c^	1 (11.1)	0 (0)	0 (0)
Gamification	1 (11.1)	0 (0)	0 (0)
UI^d^/UX^e^	1 (11.1)	0 (0)	0 (0)
Degree			
Doctoral degree	3 (33.3)	2 (22.2)	0 (0)
Master’s degree	0 (0)	0 (0)	0 (0)
Medical degree	0 (0)	1 (11.1)	0 (0)
College degree	0 (0)	0 (0)	3 (33.3)

a LGBTQ+: lesbian, gay, bisexual, transgender, queer, and other.

b MSM: men who have sex with men.

c mHealth: mobile health.

d UI: user interface.

e UX: user experience.

### Procedure

Selected experts were initially contacted by email and received an invitation describing the purpose of the study, the goals of the expert panel, and a brief overview of the MY-Hero app. Those who expressed interest and agreed to participate (N=9) subsequently received a follow-up email with several proposed dates and were asked to select a convenient time for participation. Each panel comprised experts from the relevant disciplines, and all panels were conducted between May and August 2023. All the expert panel sessions were conducted online via Zoom (Zoom Video Communications, Inc) to facilitate participation across geographic locations. Each session followed a standardized discussion protocol and a core set of questions, with slight variations tailored to each panel’s area of expertise and experience. Each panel lasted approximately 90 minutes.

At the beginning of each session, participants were introduced to the HMP mobile platform through a live demonstration of its key features and functionalities (see Multimedia Appendix 1). This demonstration was followed by a series of discussion questions developed based on the IBM model. This approach ensured that the discussion addressed the 5 core components of the IBM that affect HIV prevention behaviors (ie, affective motivations, behavioral skills, capacity, perceived and actual environmental constraints, prior habits, and salience of the behavior). Multimedia Appendix 2 presents the questions and how each question aligns with the IBM components. For each expert panel, we tailored the questions accordingly depending on their area of expertise. For example, technology and design science experts received questions emphasizing data privacy and security, as mHealth apps are regulated by the Health Insurance Portability and Accountability Act (HIPAA), and privacy and security, as well as usability considerations relevant to mHealth apps. Health science experts received questions tailored to HIV prevention strategies, implementation science, and HIV-related comorbidities. Finally, local community experts received questions related to sociocultural factors, stigma, and community-specific barriers and facilitators relevant to HIV prevention among YMSM in Malaysia.

### Ethical Considerations

This study was approved by the Yale University Human Research Protection Program and Institutional Review Board (protocol number 2000032375). All experts were required to provide informed consent before participating in the study. After the study details, including its purpose, protocol, expectations, and potential risks and benefits were explained, verbal informed consent was obtained. After each panel concluded, the recorded video and automated transcript were downloaded from Zoom. All data were then anonymized, deidentified, and stored in Yale Secure Box, a HIPAA-compliant cloud platform accessible only to authorized staff through Yale Central Authentication Service. The platform is protected by strong firewall controls and monitored by the Yale Information Security Office. Participants did not receive monetary compensation for their participation.

### Data Analysis

Each expert panel discussion was automatically transcribed verbatim using Zoom. The transcripts were reviewed against the recordings for accuracy and cleaned prior to analysis. Data were analyzed using a hybrid inductive-deductive thematic analysis approach. Two authors (JAW and XZ) independently read all transcripts multiple times to familiarize themselves with the data. Initial open coding was conducted using an inductive open-coding process in NVivo (Lumivero), where codes were generated directly from the data without imposing predefined categories. The 2 analysts then met to compare codes, resolve discrepancies through discussion, and consolidate them into a preliminary coding framework.

Following this inductive phase, the coding framework was examined through the lens of the IBM model. Specifically, codes and emerging themes were reviewed and organized in relation to the IBM constructs that guided the expert panel discussion questions (Multimedia Appendix 2). Themes related to knowledge and awareness of HIV prevention behaviors were mapped to information-related components (eg, salience of behavior), themes related to emotional engagement and motivation toward prevention behaviors were mapped to motivation-related constructs (eg, affective motivation), and themes concerning user capability and practical use of HIV prevention tools were mapped to behavioral skills and capacity and environmental constraints. This process allowed the analysis to remain grounded in participants’ insights while ensuring that the resulting themes were theoretically aligned with the IBM framework.

Codes were subsequently grouped into broader themes through an iterative process of comparison across all 3 panels. A codebook, including theme definitions and exemplary quotations, was developed and refined throughout the analytic process. The final themes represented areas of convergence across panel discussions as well as unique contributions from specific expert groups (ie, health sciences, technology, and design sciences, and LGBTQ+ community experts). Thematic saturation was assessed across panels by examining the extent to which new concepts continued to emerge. After analysis of the third panel, no substantively new themes were identified, and discussions largely reinforced and elaborated on previously identified themes. Therefore, we determined that thematic saturation was reached, and no additional panels were convened. This decision was based on the observed thematic redundancy and the adequacy of thematic depth in addressing the study aims [73].

## Results

### Overview

Thematic analysis yielded 3 key domains. The first domain, implementation science, highlights factors influencing the adoption and sustained engagement with the MY-Hero app among Malaysian YMSM. The second domain, design science, addresses UI, gamification, and technological enhancements to optimize UX. The third domain, the Malaysian context, focuses on culturally specific considerations, such as privacy and security concerns, that are critical to the app’s relevance and acceptability. Together, these domains capture the essential components needed to ensure the effectiveness of the MY-Hero app. Table 2 presents the 3 major domains and subthemes under each domain.

**Table 2.** Three identified domains, their subthemes, and alignment with the integrated behavioral model (IBM) constructs.

Identified domains and subthemes		IBM constructs				
		Affective motivations	Behavioral skills and capacity	Perceived and actual environmental constraints	Prior habits	Salience of the behavior
Implementation science						
Clinical affiliation			*✓*	*✓*	*✓*	*✓*
Harm reduction			*✓*	*✓*	*✓*	*✓*
The rule of simplicity		*✓*				*✓*
Innovative strategies to increase the reach		*✓*	*✓*			
Design science						
Potential adverse effects of competition		*✓*				*✓*
UI^a^ improvements		*✓*	*✓*		*✓*	
Concerns on in-app user interactivity		*✓*		*✓*		
Enhancing gamification elements		*✓*	*✓*			*✓*
The Malaysian context						
Contextualization				*✓*		*✓*
Data security and privacy		*✓*		*✓*		*✓*
Harnessing customization		*✓*				*✓*

a UI: user interface.

### Domain 1: Implementation Science

Our expert panels emphasized several strategies that can increase broader adoption of the MY-Hero app among Malaysian YMSM, as well as features that can increase its effectiveness in improving HIV prevention behaviors.

#### Clinical Affiliation

Integrating the app within the existing clinical settings, such as enabling users to directly connect with health care providers, may increase app adoption. Features such as schedule management for HIV care and one-on-one mental health counseling would position MY-Hero as a comprehensive, one-stop platform for addressing the health needs of this population. This integration is particularly relevant given the high levels of discrimination and internalized stigma faced by this population in traditional health care settings. Seamless, app-based access to providers can reduce reliance on potentially stigmatizing face-to-face encounters and lower barriers to engaging in HIV prevention services.

*It’s like they (Malaysian YMSM) are opportunistic, so their motivations of seeking help is up and down. So, if we don’t have those services (clinical affiliation) ready there in the app, and well, these people would just stop there. They will be just at the pre-contemplation stage, like thinking that I should have to change, I should actually go get PrEP, but it is just thinking on it. They will never come to the actions. Once they come to the actions, we must have a service built in the app that’s already there for them*.

[AY, Health Science Expert]

#### Harm Reduction

Experts recommended incorporating features within the app to address the growing need for harm reduction services among Malaysian YMSM, who are experiencing rising rates of substance use, a key antecedent to HIV risk behaviors [20,74,75]. Suggested enhancements include embedding harm reduction education and information content (ie, substance use and addiction support) within the app’s multimedia content library, as well as enabling users to order harm reduction supplies from the app. These additions could further strengthen the app’s role in supporting holistic HIV prevention efforts.

*We observe that they most commonly want information or advice about fundamental aspects of harm reduction. And it’s about people not looking to be told “Don't do it.” Usually what they’re seeking online is advice about how to do so more safely, how to do so in a way that doesn’t complicate their sex life or have an adverse impact on the other aspects of their everyday life, about the drugs that shouldn’t be taken in certain combinations and about managing come down.*

[AB, Health Science Expert]

*So, if I think about a big site here in [blinded for review] that we were doing some analysis not long ago. On a Tuesday or a Wednesday, when people are most likely to be experiencing a comedown after using crystal methamphetamine, there is a massive spike in traffic to web pages focused on managing a crystal meth related comedown. There’s a lot of return traffic, with users repeatedly revisiting those pages over time. So it just might be worth of introducing harm reduction information, because it’s something that it’s very widely sought after, and can be quite hard to identify in in many contexts, particularly in some more socially conservative environment*.

[AB, Health Science Expert]

#### The Rule of Simplicity

Our goal was to create a mobile “one-stop shop” model for Malaysian YMSM to address their HIV-related and comorbidity-related health needs (eg, mental health, substance use, and chemsex). To this end, the MY-Hero app includes a range of integrated features adapted from the HMP platform. One expert, however, emphasized the importance of simplicity, advocating for a “less is more” approach. Rather than aiming for a comprehensive solution, the app should prioritize achievable goals directly related to HIV prevention. Including too many features may introduce unnecessary complexity and overwhelm users, ultimately reducing engagement. Streamlining MY-Hero’s functions may therefore improve its usability and adoption among Malaysian MSM.

*I’m not sure what exactly the main purpose of this app is. As a user, should I use it for education or something else? So I’m not sure whether there’s a site map that you can look at it. And then when you go down the features, they ask for sexual behaviors and all that. That is very sensitive. Why does the app ask about this? It’s only when you (the moderator) mapped this with the PrEP calendar that I realized why the app ask for sexual behavior information. This connection was not obvious when you (the moderator) first opened the app. So, what is this app is for? What can users do with it? Why certain data are collected*?

[CJ, Technology Science Expert]

#### Innovative Strategies Are Needed to Increase the Reach of MY-Hero

To increase the acceptance and adoption of the MY-Hero app, our community experts emphasized the need for innovative outreach strategies, including partnering with local LGBTQ+ community leaders, using trending social media platforms, and engaging users through gaming platforms like Discord (Discord Inc). Strategies also include reaching Malaysian YMSM in physical venues they frequently visit, such as clubs, bathhouses, etc. Additionally, given that there are several other mHealth apps for Malaysian MSM [76,77], it is important to differentiate the MY-Hero app from these existing apps.

*If you can identify where your target population hangs out, such as coffee shops, nightclubs or online platforms that they frequently use, that could support more targeted outreach. Since you are targeting YMSM, I would suggest Discord, a social platform that allows instant communication among gamers. You can place a flyer with a QR code. All they have to do is to scan the QR code, and it will automatically open the downloads page on app store so they can download it. And you can incentivize this as well. For example, if it is a coffee shop, instead of sending them a water bottle, you can offer a free coffee when they download the app through that QR code. This could create a targeted campaign that helps increase downloads of your application*.

[ED, Technology Science Expert]

I’m also thinking if there is a way for the project to engage the business owners, people who own clubs, and karaoke. They can encourage to provide promotion vouchers. We should promote on a regular basis. People are motivated to come on to win some of the prizes. They can either win, or they could be the first 100 to get it. That kind of gimmicks might work.

[RT, Malaysian LGBTQ+ Expert]

### Domain 2: Design Science

#### Potential Adverse Effects of Competition

While gamification elements, such as competition and rewards, can increase user engagement and promote positive health behaviors [34], their design requires careful consideration. Experts suggested that competition and rewards should be designed to reinforce positive health behaviors, such as frequent condom use, rather than merely increasing app use or behavior tracking itself. Given that MY-Hero addresses multiple health domains, including substance use, smoking, and condomless sex, gamified features such as leaderboards or rewards tied to the quantity of tracked behaviors may be counterproductive. This design approach risks unintentionally encouraging engagement in harmful behaviors simply to increase the number of logged entries and receive incentives or recognition. Therefore, gamification should be structured to promote healthy actions without inadvertently encouraging risk-taking.

*I think there is a risk that it (competition or leaderboard) has an inhibiting effect, because you see yourself lower on that leaderboard, and that can have an impact in them. But if it (leaderboard) is a composite of a few different domains, the likelihood that one person is performing poorly across all areas is reduced. You can be saying, oh, you’re doing really well in this category, but think about how you could be raising your game over here or having to think about (another category). You know what you can be focusing your attention on next time.*

[AB, Health Science Expert]

*The problem is when we introduce leaderboard, we introduced competitions as well. When in a competition, someone it’s a winner, and someone had to be a loser. Is this a message that we want to bring to our audience? Compared to competing with others, I think there are a lot of strength to competing with oneself over time instead.*

[AY, Health Science Expert]

Some existing gamification elements can also be further enhanced to promote healthy competition. For example, one expert suggested adding “stacking rings” to user avatars as a form of vanity rewards or progression symbol, similar to Apple Watch activity rings. Each ring would represent progress in a specific behavior and signal status and achievement to other users. For example, a red ring could indicate the number of HIV testing kits ordered per month, while a blue ring could represent the number of PrEP taken per month.

*You know this Apple Watch thing that that I just started wearing because I’m trying to improve my own health. and it’s got these rings on it. You know these rings that it has 3 different rings. You know you could be good in one ring, but maybe we can another*.

[AB, Health Science Expert]

#### User Interface Improvement

The experts identified user interface (UI) elements that can enhance the UX, including interactive avatars and haptic or animated feedback. In the current version of the MY-Hero app, avatars are static and customizable, with unlockable facial features, clothes, and accessories tailored to the Malaysian context. One expert suggested making avatars interactive, with motions, gestures, and non-verbal expressions, could enhance user engagement by making them feel more human-like [78]. Additionally, digital interactions like haptic feedback (eg, vibrations) or animated responses (eg, fireworks upon completing a task or reaching a milestone) can foster a stronger sense of presence and immersion, providing a more realistic experience and ultimately improving sustained app engagement.

*I think you can enhance your avatars. You already use rewards as a gamification technique in your app. You can add a circle around avatars if they use the app for 10 hours, let’s say, or if they contribute something using the app. For someone who use the app for a hundred hours, you can add another circle, or create a moving rainbow around their avatar. That means, when I look at someone’s avatar, I can say that the person is contributing less time or more than me, and I can try to contribute more to have all these (circles) to have a fancier avatar*.

[ED, Technology Science Expert]

*I’m a Google map contributor. In Google, my avatar shows, I’m a 6-star user. If you could do something like that, that’d be nice to motivate someone to complete task and then become a higher person (app user*).

[CH, Technology Science Expert]

#### Concerns on In-App User Interactivity

Allowing users to interact with each other within the app can foster a sense of relatedness, which can lead to more information exchange and emotional support [79,80]. However, one expert stated that such interactive features can be misused for social networking or dating purposes, potentially shifting the app’s focus away from HIV prevention and managing comorbidities. This may also raise Malaysian YMSM’s concerns about privacy and the potential for sensitive information to be inadvertently disclosed.

If you have the feature to talk to other people, there’s definitely great engagement that people can get to know each other. But I think when once we open that threshold, a lot more problems can also come with it. For example, people can harass each other like people do online. All these things will also come in and requires moderation and guidelines. All these things can also be a bit too much. This goes back to what is the purpose of this app. Building a community online is a very different goal as compared to increasing people’s access to health or management of sexual health. It really goes back to what the purpose of this app is to decide whether opens up for that function (interactivity) or not.

[GC, Malaysian LGBTQ+ Expert]

#### Enhancing Gamification Elements

Experts also suggested the incorporation of progression systems like a progress bar or a level-up system that visually represent users’ advancement toward their goals. These features can foster a sense of accomplishment and motivate continued engagement on their HIV prevention journey. Additionally, such progress systems support self-monitoring, a key behavioral change technique that has shown high effectiveness in increasing PrEP uptake, HIV testing, and ART adherence [81,82].

There are so many things in the app. Obviously, we want them (Malaysian YMSM) to learn everything. On the landing page, (it would be helpful if) we put a progress bar to indicate how much they (Malaysian YMSM) have learned based on their past app activity. Progress makes people feel that they want to get to complete, so that it (a progress bar) will force people to learn and finish up the app use. When you open the app it (a progress bar) is showing. It’s like 98%.

[CH, Technology Science Expert]

Another gamification element recommended by our experts was to incorporate the element of surprise. Unexpected rewards, spontaneous challenges, or newly introduced app features can spark curiosity, excitement, or a sense of uncertainty, which all can foster greater user engagement.

*Maybe you can incorporate the element of unlocking new things. Why people keep playing candy crush? It is because they want to see what’s next. They are engaged like addicted to it. The element of unlocking new things or unlocking a surprise is something we will drive people to keep using the app. People like the element of surprise*.

[CH, Technology Science Expert]

One expert also suggested that elements like narrative stories can also be a more fun, engaging way for Malaysian YMSM to gain health information. Unlike traditional learning through quizzes and reading, narrative stories improve learning through gamified characters and a story plot that can increase knowledge retention.

*Maybe you can add storytelling to your app. As people use your app, they can progress through the story. It (storytelling) will improve your app and make it different than other similar apps*.

[ED, Technology Science Expert]

### Domain 3: The Malaysian Context

#### Contextualization

Our experts emphasized the importance of culturally and contextually tailored app content to ensure high app engagement and to meet the needs of our targeted population. For example, tangible rewards such as vouchers, coupons, and gift cards should be linked to stores and services commonly used by Malaysian YMSM. Customizable avatars can also include Malay clothing and accessories. Two experts also emphasized the importance of adding Mandarin, one of Malaysia’s 3 most spoken languages, to the app’s language options, which currently include only Malay and English.

*The rewards will have to be local to fit to what the youngsters like. I think Starbuck will be much more appealing for them and also tea leaf (The Coffee Bean & Tea Leaf* *Malaysia”*. *It’s a small reward that can drive them. These people they usually would love to have, a Starbucks or something similar to it.*

[AY, Technology Science Expert]

*What languages do the app support right now? How about Mandarin?*

[RV, Malaysian LGBTQ+ Expert]

#### Data Security and Privacy Concerns

Our experts also identified security and privacy concerns as potential barriers to adopting the app. Given that the app contains content that could suggest same-sex activity, which is criminalized in Malaysia, users may fear potential legal consequences. To address this, it is essential to reassure users that their data are protected through robust security measures, such as encryption and secure user authentication protocols.

*I think the users might raise some concerns about those privacy issues*.

[CJ, Technology Science Expert]

*Where would this app be located? And how do we handle privacy and data protection? There’d be a whole lot of other things to do that*.

[RT, Malaysian LGBTQ+ Expert]

#### Customization

Customization allows users to modify the app content to personal preferences, resulting in a more relatable and engaging interface [83]. By enabling users to control how the app is used, how features are presented, and how to integrate the app into their daily life, we can provide a sense of autonomy and ownership, which can enhance sustained app engagement [84]. While the MY-Hero app offers avatar customization, experts suggested expanding customization to include personal goal setting and milestone tracking to better support autonomy and engagement.

*Is this a standard milestone or that milestone that you can customize? (I asked this) because different people might have different types of milestones. If you make it a bit more customizable, it can also let you understand the behavior (app use, HIV prevention behaviors) of the users*.

[RV, Malaysian LGBTQ+ Expert]

## Discussion

### Principal Findings

The aim of this study was to use an interdisciplinary expert panel strategy to identify key features in a gamified mHealth app, MY-Hero, for HIV prevention and comorbidity management among Malaysian YMSM. The expert panel identified several themes, including (1) establishing clinical affiliation and linkage to HIV testing, PrEP, and related services, (2) enhancing UI and UX by optimizing usability and interactivity, (3) incorporating customizable health content, tailoring interventions based on individual profiles and preferences; and (4) leveraging certain gamification elements carefully, such as contextualized reward systems and progression tracking, to reach high adoption and sustained engagement. The panel also highlighted (5) the importance of privacy and data security in the Malaysian context. Given that the Malaysian YMSM often experience substantial stigma and discrimination that pose barriers to their access to HIV prevention care and treatment [1-3], along with being disproportionately affected by medical and psychiatric comorbidities, like cigarette use [10], alcohol use, and methamphetamine use [6,11,12], depression [13], and STIs, including syphilis [15-17], an innovative, accessible, and culturally responsive gamified mHealth app, such as the MY-Hero app, offers a promising opportunity by providing a safe, confidential, and engaging platform where YMSM can address their diverse health needs.

The MY-Hero app was developed from the HMP platform with a range of core, established features, including HIV testing information and the ability to order home kits, a medication tracker, and other health-related tools. Following the results, we made several improvements in the MY-Hero app. Multimedia Appendix 3 includes implementation details for each gamification element, including how each gamified feature is operationalized in the app, the mechanism through which it is triggered, the type of reward or feedback provided (eg, virtual points, badges, or content unlocking), and the types of user behavioral data tracked to support these features (eg, module completion, quiz responses, and engagement metrics). Multimedia Appendix 4 presents screenshots of the MY-Hero app illustrating its main features and UI, including how users interact with the app, app navigation structure, gamified features, and other educational features.

These improvements were implemented and prioritized based on four guiding principles, including (1) feasibility with the technical infrastructure and resource constraints, (2) cultural and contextual relevance for Malaysian YMSM, (3) impact on HIV prevention and comorbidity management. Identified subthemes that directly influence user privacy and security, HIV prevention and comorbidity efforts were prioritized for immediate implementation (ie, clinical affiliation and enhanced password protection), and (4) within the scope of the work, which was to design an independent end-user mHealth app to support Malaysian YMSM’s self-management on HIV prevention and comorbidities. Suggestions that could enhance user engagement were integrated while balancing concerns about unintended competitive effects, such as leaderboard modifications and interactivity between users. Design simplification, including reducing unnecessary features and improving navigation, was also prioritized to improve usability.

One of the major themes was user privacy and security. The MY-Hero app was hosted on the HMP server, and the security for the HMP platform operates at both the app and server levels to ensure robust protection of user data. At the app level, users authenticate through the OpenID Connect (OIDC) framework built on OAuth 2.0 (Open Authorization 2.0), with secure login mechanisms including passwords and mobile biometric authentication (FaceID/TouchID), and access is managed through role-based permissions. At the server level, the platform uses a multilayer architecture (proxy, application, and database), with each study hosted on a dedicated server and database instance within Florida State University’s secure data center, where encrypted storage protects data at rest. Data are also encrypted during transmission through enforced HTTPS protocols, and additional protections include restricted database access, routine security patching, and nightly encrypted backups. Following the experts’ recommendations, we further strengthened privacy protections by allowing users to register with pseudonyms, encrypting email addresses, and not requiring real names when ordering HIV testing kits or redeeming swags. To support user interactivity while maintaining anonymity, the platform only displays limited profile information, such as avatars and aggregate in-app activity indicators (eg, number of likes, comments, or posts). No personally identifiable information is visible to other users. These measures were implemented to minimize risks to privacy and confidentiality, which are particularly important given the sensitive legal and social context surrounding same-sex behavior in Malaysia. By strengthening privacy protections, these design choices may also increase users’ trust in the platform, encourage greater self-disclosure and information exchange, and ultimately enhance user engagement and the app’s overall effectiveness [85].

Two recommendations were not implemented, including linkage to care providers outside of the study team (suggested by expert AY) and creation of separate leaderboards for individual in-app activities (suggested by expert AB). These were not feasible because they were outside the scope of the study and constrained by budget and time limitations. Additionally, activity-specific leaderboards, particularly those related to sensitive activities such as sexual behavioral tracking, were not adopted because they could introduce privacy risk by potentially exposing users’ confidential information and jeopardizing their safety, especially in a context where same-sex is criminalized in Malaysia. Table 3 summarizes the enhancements in MY-Hero based on study results, the design domains, and recommendations that were not implemented, along with the rationale for those decisions.

**Table 3.** Translation of expert panel results into design domains, implemented enhancements, and unimplemented recommendations.

Results	Design domains	Enhancement actions taken in MY-Hero	Unimplemented recommendations and rationale
Clinical affiliation	Clinical integration	Added an appointment feature where users can connect with a care provider on the study team for counseling, appointment scheduling, and direct messaging regarding health concerns	Linkage to care providers outside of the study team due to the budget constraints and limited duration of the study period; being the study scope, an app that links YMSM^a^ and clinical providers will be explored in future studies
Harm reduction	Influencer outreach	Added content related to harm reduction in multimedia content library; included harm reduction supplies like condoms into the party surprise pack	—^b^
The rule of simplicity	Feature simplification	Reduced features like goal setting and milestones	—
Innovative strategies are needed to increase the app reach: collaborating with local NGOs^c^	App marketing	Reached out to 12 LGBTQ+^d^ influencers who have over 50,000 followers on Instagram or TikTok	—
Competition and rewards can affect YMSM’s health motivation	Engagement optimization	Added a leaderboard and points system that rank order users based on users’ HIV testing kit ordering frequency and in-app activities (eg, comments, likes with other users, reading multimedia library content); redeemable swags such as water bottles and power banks	No separate leaderboards for each in-app activity due to budget constraints. Activity-specific leaderboards (eg, for sexual behaviors tracking) could inadvertently expose sensitive user behaviors
UI^e^ improvement	Usability enhancement	Adjusted the background color to be automated adaptation to day and night time	—
Navigation		Simplified the number of screens and paths that users need to go through from one feature to another	—
Disagreement on interactivity among experts	User interactivity enhancement and tailoring	Limited the one-on-one interactivity between users only on public forums; added private, limited interactivity where users can follow each other but can only see the number of likes and comments of the followed users	—
Enhancing gamification elements	Contextual tailoring	Provided random party surprise packs to participants	—
Content tailored to Malaysia	Cultural tailoring	Contextualized avatars with traditional Malaysian clothing styles, on-trend accessories in Malaysia	—
Data security and privacy concerns	User privacy and security	Enhanced privacy and security protection, that is, pseudonymous registration with encrypted email addresses, no real-name requirement; anonymous interactions	—
Harnessing customization	Customization enhancement	Allowed users to pick their own goals based on their health needs	—

a YMSM: young men who have sex with men.

b Not applicable.

c NGO: nongovernmental organization.

d LGBTQ+: lesbian, gay, bisexual, transgender, queer, and others.

e UI: user interface.

### Interpretation of Findings and Their Linkage to the IBM Model

A central theme was the establishment of clinical affiliation and provision of inclusion of harm reduction services to improve access to HIV care and treatment within the app. This aligns with prior research indicating that mHealth apps are more effective when linked to offline care pathways [86,87]. In the context of HIV, mHealth interventions linked to offline services, such as clinic scheduling, telehealth PrEP models, and provider messaging systems, produce greater improvements in HIV testing, retention in care, and medication adherence compared to standalone informational apps [88-90]. Conversely, studies of gamified or informational apps without integrated care pathways report more modest or short-lived behavioral effects, underscoring the importance of structural linkage in sustaining HIV prevention behaviors. By enabling appointment scheduling and direct communication with Malaysian health care providers and the ability to order harm reduction supplies within the app, this platform can help mitigate many barriers faced by Malaysian YMSM, including stigma and discrimination in health care settings [91]. Aligning with multiple components of the IBM model, clinical affiliation can provide many benefits. Specifically, it reduces perceived and actual environmental constraints by minimizing stigma-related barriers in traditional face-to-face settings. It also enhances behavioral skills and capacity by simplifying the process of accessing care and increases the salience of the behavior by embedding HIV-related health actions within a trusted digital platform. Furthermore, by creating positive experiences in health care engagement in a gamified app environment, it may help disrupt prior negative habits and build affective motivation toward sustained HIV prevention behaviors. Despite being unable to establish clinical affiliations outside of the study team due to the project’s scope, time, and budget constraints, we enhanced the app by incorporating the appointment feature that allows users to connect with clinical providers within our study team for counseling, appointment scheduling, and direct messaging for health concerns. In future work, we plan to explore how integrating direct communication with care providers in Kuala Lumpur may further support and improve health behaviors among Malaysian YMSM.

Another finding was the need to collaborate with local nongovernmental organizations (NGOs). This process will require not only software developers but also partnerships with health care providers for service integration and possibly local stores for reward sponsorship. Prior research among MSM populations indicates that trust in HIV-related mHealth interventions depends not only on app functionality but also on who develops, endorses, and governs the platform. For example, studies among Malaysian MSM have shown that users value transparency regarding app ownership and management and perceive endorsement by reputable NGOs or health authorities as enhancing credibility and quality assurance [92]. At the same time, these studies also highlight concerns about government access to personal data and potential surveillance in criminalized settings, suggesting that institutional involvement must be paired with robust privacy protections and transparent governance structures to maintain user trust. In the Malaysian context, one solution could involve early engagement with the Ministry of Health in Malaysia and local NGOs to establish clinic linkages and content accuracy. The Malaysian Ministry of Health endorsement or involvement could lend credibility and help in scaling the app to reach the intended audience. By positioning the MY-Hero app as a credible and trusted resource, especially if managed by a reputable NGO or research institution in partnership with the Ministry of Health, it can strengthen Malaysian YMSM’s affective motivations and trust, which are critical in a context where fear of surveillance or discrimination is prevalent.

Our findings also emphasized the importance of UI and UX enhancements in driving engagement and sustained use. Experts from design and health sciences emphasized that an intuitive interface is essential for user satisfaction, highlighting the importance of simplicity, streamlined navigation*,* and enhanced customization. Customizable avatars, interactive feedback (haptic or animated responses), and progression systems were also suggested. Gamification elements were also recognized as a pivotal feature of the app. Experts advised on the careful design of competition and reward systems to reinforce positive health behaviors rather than incentivizing risk behaviors. This reflects broader findings in gamification literature, where properly designed game mechanics can enhance motivation and behavior change, particularly in public health interventions [93-102]. This also provides a theoretical insight, in that interventions must temper generic behavioral techniques with context-specific psychosocial factors. Research on gamified health interventions indicates that competitive elements can produce adverse effects on users’ motivation and engagement when poorly matched to the context or user needs [103,104]. For example, studies have documented potential unintended side effects (ie, reduced health motivation) of gamification in health behavior change systems [105]. Likewise, research on competitive gamified learning found that simple competition was often disengaging for or rejected by many users [104]. Finally, analyses of leaderboard effects in health tracking show that competitive mechanics may benefit some user subgroups while harming others, underscoring the importance of tailoring such strategies to the specific audience and setting [103]. Particularly in stigmatized contexts, the incorporation of competition needs to be tailored and designed carefully.

Additionally, the concept of surprise elements, such as unlocking new features or rewards, can maintain long-term engagement, a strategy that has been successfully implemented in commercial gaming apps [106]. The inclusion of unexpected rewards positively influences user motivation and engagement by disrupting routine interaction patterns and generating new interest [107]. Such disruption can stimulate anticipation cycles, thereby increasing engagement intensity and encouraging continued participation over time [108]. Moreover, instructional design literature highlights that controlled unpredictability activates curiosity, one of the core intrinsic motivational drivers, prompting users to remain engaged in order to discover what comes next [109]. Our experts highlighted that such surprise can be particularly important for YMSM, who respond positively to new and surprising content. In HMP, the rewards were only virtual badges. Building on our experts’ suggestion, we expanded this gamification element by providing tangible, redeemable rewards such as T-shirts, water bottles, power banks, and a surprise party pack that includes harm reduction items, such as lube, condoms, fun sexual activities, or games. All these aforementioned suggestions on UI and UX align with the behavioral skills construct in the IBM model in several ways. First, interactive and personalized features, improved navigability, and more customization and simplicity can enhance affective motivations by making the app more enjoyable, user-friendly, and emotionally resonant. Progression systems and intuitive design support users’ behavioral skills and capacity by making tasks easier to understand and accomplish. Features such as surprise elements and visual progress tracking increase the salience of HIV prevention behaviors by reinforcing users’ attention and engagement. Furthermore, by building new health habits through repeated in-app interactions, these elements can help replace prior disengagement with the proactive health behavior. To maximize the effectiveness of these improvements, our findings suggest that they must be implemented with careful, context-specific design considerations.

Concerns about data privacy and security were identified as a critical theme, reflecting their heightened relevance within the Malaysian context. Community experts highlighted that fear of data breaches and privacy violations could deter YMSM from engaging with the MY-Hero app [26]. This concern is consistent with prior research among Malaysian MSM, where cross-sectional findings indicate that privacy, confidentiality, and data security concerns are significant barriers to willingness to use HIV-related mHealth apps, directly influencing trust and uptake [26]. Similarly, qualitative focus group studies with Malaysian MSM have identified fear of third-party data access, uncertainties surrounding data storage and management, and broader ethical concerns about confidentiality as prominent factors shaping app acceptability [26,92]. Beyond the Malaysian context, reviews of digital health technologies have consistently identified privacy and confidentiality as persistent ethical challenges in eHealth and mHealth interventions, underscoring the importance of robust safeguards to support sustained participation [110,111]. Moreover, digital privacy risks are particularly consequential for marginalized populations in settings where same-sex behavior is criminalized, as data breaches may exacerbate stigma, discrimination, or legal vulnerability [26,112]. Thus, strong security measures, including end-to-end encryption, anonymized user data, and transparent data governance policies, are essential for fostering trust and encouraging app adoption. See Table 2 above that illustrates the linkage between each theme and each of the 5 components of the IBM model that are important to enhancing HIV prevention behaviors.

### Importance of Contextualization in Malaysia

The findings highlighted several design principles that are important and unique to the Malaysian context. These included collaborating with local NGOs to enhance the reach of the MY-Hero app, adapting UI to reflect Malaysian cultural identities through customizing avatars and simplified UI, carefully designing leaderboard and interactivity features to avoid unintended consequences, and enhancing data privacy and security. These improvements were made in direct response to the Malaysian sociocultural and legal context, particularly the criminalization and stigma that this vulnerable population experiences.

This contextualization has both theoretical and practical implications. First, it suggests that the IBM model may benefit from the incorporation of a sixth construct, that is, contextual consideration. While each existing 5 construct addresses key determinants of health motivation and behaviors, our findings demonstrate that sociocultural, structural, and legal contexts fundamentally shape how those constructs operate in practice. Integrating contextual considerations can enhance the scalability, cultural appropriateness, and feasibility of interventions tailored to specific populations. The contextualization we made in the MY-Hero app also distinguishes the app from previous Western mHealth interventions by adding cultural specificity that is important to countries in Southeast Asia.

Practically, the findings underscore the importance of culturally balanced design in multicultural settings. Malaysia’s diverse population, including Malay, Chinese, and Indian communities, needed careful consideration of features and UI to ensure its acceptability, reach, and scalability. In this way, MY-Hero can serve as a skeleton for adapting mHealth interventions to other Southeast Asian countries facing similar structural and cultural dynamics. More broadly, these findings can inform the design and implementation of HIV-related mHealth interventions in other multicultural countries with concentrated epidemics, such as South Africa [113], Thailand [114], and Brazil [115,116], where cultural diversity and stigma similarly shape intervention uptake. Finally, several recommendations were not fully implemented due to budget and timeline constraints, as well as privacy considerations. By balancing expert recommendations with what is doable, this study shows how mHealth interventions should be adapted to align with real-world implementation constraints and the needs of vulnerable populations.

### Strength of the Study

A key strength of this study was the use of an interdisciplinary expert panel leveraging both scientific evidence and community insight. This approach allowed us to generate a rich, contextually grounded set of recommendations relatively quickly, without the logistical and ethical complexities of conducting large-scale end-user workshops in a stigmatized population. The inclusion of community experts alongside data and health scientists helped ensure that the features were not only theoretically sound but also pragmatically relevant and respectful of Malaysian YMSM’s lived realities.

Additionally, our approach deliberately linked each identified theme to the IBM model. This theoretical grounding can help in later evaluating the app’s mechanism of action. We can hypothesize, for example, that the avatar and reward system will enhance affective motivation, leading to greater intention to test for HIV, which in turn (with easy service linkage) results in higher testing uptake. Additionally, grounding the discussion in the IBM gave the analysis a structured lens, which is a novel aspect of gamification design, as many design processes might not explicitly reference theory during feature brainstorming.

Another strength is the novelty of our focus. To our knowledge, this is one of the earliest attempts to outline a comprehensive gamified mHealth solution specifically for HIV prevention and comorbidities in Malaysian YMSM. While several mHealth interventions have been developed in Malaysia to support HIV prevention, such as the JomPrEP (University of Connecticut) app, MY-Hero differs in several important ways. For example, JomPrEP primarily focuses on facilitating PrEP awareness, access, and linkage to services through informational resources and service navigation [77,78]. In contrast, MY-Hero is a gamified “one-stop shop” for Malaysian YMSM to address not only HIV prevention efforts but also comorbid health needs, such as mental health and substance use. Additionally, by incorporating a diverse set of gamification elements, MY-Hero aims to move beyond information delivery toward a more fun, engaging, and sustained behavior change approach within the Malaysian HIV prevention landscape. It addresses a clear gap in the literature and local practice, as current HIV programs in Malaysia have yet to fully harness gamified mHealth, despite the substantial needs [68].

### Limitations

There are several limitations to acknowledge. Our expert panel did not include many actual end users (ie, Malaysian YMSM themselves), apart from 3 community representatives who had insight into the community’s needs. This approach was intended to minimize potential risks to YMSM participants in a study setting given the social context, but it means some user preferences might not have been fully captured. For instance, while experts assumed avatars would be popular, it is possible some YMSM might find them trivial or childish; only direct user testing can validate these assumptions. We mitigated this by relying on community experts’ knowledge and literature on user preferences. Future research should focus on pilot-testing MY-Hero with Malaysian YMSM to evaluate usability and its impact on HIV prevention behaviors and comorbidities. Second, experts were recruited using convenience sampling, which may introduce selection bias and limit the diversity of perspectives represented in the panels. Although we attempted to reduce this risk by applying strict eligibility criteria, including extensive professional experience, demonstrated expertise in relevant fields, and familiarity with HIV prevention or gamification, our panel may still reflect the viewpoints of a specific network of experts. Future research could incorporate a broader range of stakeholders, including additional community representatives, implementation partners, and end users, to further refine the intervention design and ensure broader representativeness.

### Conclusion

By integrating expert insights from health care, technology, and community perspectives, this study highlights several key principles for the design of gamified mHealth interventions for HIV prevention among young and marginalized populations. First, interdisciplinary collaboration is essential for developing interventions that are clinically and theoretically grounded and responsive to the needs and preferences of the target users. Second, carefully designed gamification elements, including rewards, customization, and surprise, can enhance user engagement and health motivation, while some elements like competition require thoughtful and careful implementation to avoid unintended negative effects. Third, cultural adaptation and tailoring to contextual factors, including trust, privacy, and partnerships with local community organizations, are critical for ensuring acceptability and sustained engagement of mHealth interventions.

The findings demonstrate how gamification elements can be strategically aligned with behavioral targets from the IBM to support HIV prevention efforts. Beyond informing the development of the MY-Hero app, this study highlights transferable design principles and a replicable interdisciplinary development process that can guide the creation of scalable, culturally responsive gamified mHealth interventions across diverse settings. These insights also create opportunities for future interventions that integrate emerging digital technologies, such as AI, video game-based learning, and metaverse-enabled social and immersive experiences to deliver more personalized, adaptive, and engaging health support.

## Supplementary material

10.2196/80334 Multimedia Appendix 1 Main features of the HealthMPowerment (HMP) functionalities and main features of HMP and integrated behavioral model (IBM) Constructs.

10.2196/80334 Multimedia Appendix 2 Expert panel discussion questions mapped to integrated behavioral model (IBM) key components of HIV prevention behavior.

10.2196/80334 Multimedia Appendix 3 Operationalization, trigger mechanisms, and targeted behavioral determinants of gamification elements in the MY-Hero app.

10.2196/80334 Multimedia Appendix 4 Screenshots of the main features of the MY-Hero app.

## Abbreviations

HIPAA: Health Insurance Portability and Accountability Act
HMP: HealthMPowerment
IBM: integrated behavioral model
LGBTQ+: lesbian, gay, bisexual, transgender, queer, and others
mHealth: mobile health
NGO: nongovernmental organization
OIDC: OpenID Connect
PrEP: pre-exposure prophylaxis
STIs: sexually transmitted infections
TPB: theory of planned behavior
UI: user interface
UX: user experience
YMSM: young men who have sex with men
