# Supplementary material for: Designing a Gamified mHealth App for HIV Prevention and Comorbidities Among Malaysian Young Men Who Have Sex With Men: An Interdisciplinary Expert Panel Study
Source: J Med Internet Res. 2026 Sep 8;28:e80334. doi: 10.2196/80334 (PMC13552997; doi:10.2196/80334)
Supplement: Supplementary file 1 [file jmir-v28-e80334-s001.docx]

**Appendix 1A.** Main features of the HMP functionalities.

**Appendix 1B.** Main features of HMP and IBM Constructs.

| **Main features of HMP** | **IBM Constructs** | | | | |
| --- | --- | --- | --- | --- | --- |
|  | **Affective motivations** | **Behavioral skills and capacity** | **Perceived and actual environmental constraints** | **Prior habits** | **Salience of the behavior** |
| Comorbidities | ***✓*** | ***✓*** | ***✓*** |  | ***✓*** |
| Visual Dashboard |  | ***✓*** |  | ***✓*** | ***✓*** |
| Knowledge Center | ***✓*** | ***✓*** |  |  | ***✓*** |
| Adherence Strategy |  | ***✓*** | ***✓*** | ***✓*** | ***✓*** |
| Customizable Avatars | ***✓*** |  |  |  | ***✓*** |
| Reminders |  | ***✓*** | ***✓*** |  | ***✓*** |
| Rewards | ***✓*** |  |  | ***✓*** | ***✓*** |
| Fun Activities | ***✓*** | ***✓*** |  |  | ***✓*** |
| Order HIV Testing Kits |  |  | ***✓*** |  | ***✓*** |
| Ask the Expert |  | ***✓*** | ***✓*** |  | ***✓*** |
| Health Tracker |  | ***✓*** |  | ***✓*** | ***✓*** |
