# Supplementary material for: Designing a Gamified mHealth App for HIV Prevention and Comorbidities Among Malaysian Young Men Who Have Sex With Men: An Interdisciplinary Expert Panel Study
Source: J Med Internet Res. 2026 Sep 8;28:e80334. doi: 10.2196/80334 (PMC13552997; doi:10.2196/80334)
Supplement: Supplementary file 2 [file jmir-v28-e80334-s002.docx]

**Appendix 2**. Expert Panel Discussion Questions Mapped to IBM Key Components of HIV Prevention Behavior

| **Questions Asked** | **IBM Components Addressed** |
| --- | --- |
| What specific improvements or new features could make *MY-Hero* more impactful and relevant to the HIV prevention needs of Malaysian YMSM? | *Salience of behavior*: Helps ensure HIV prevention remains top-of-mind by making the app culturally relevant. *Affective motivation*: Invites features that emotionally resonate with users, increasing engagement. *Perceived/actual environmental constraints*: Encourages addressing usability barriers, stigma, or access issues. |
| How can gamification elements be better designed or refined in *MY-Hero* to motivate Malaysian YMSM to engage in HIV prevention behaviors through? | *Affective motivation:* Gamification affects emotions like enjoyment and reward, motivating preventive behavior.  *Behavioral skills and capacity:* Features like challenges, leaderboards, and multimedia content library can build skills and confidence.  *Salience of behavior:* Games can reinforce repeated engagement, making prevention more routine and visible. |
| What strategies or design elements can be implemented to increase sustained app usage and habit formation among Malaysian YMSM? | *Prior habits:* Encourages insights on how to replace or build on existing digital habits (e.g., scrolling, gaming).  *Salience of behavior:* Push notifications, reminders, or streaks can keep HIV prevention salient over time.  *Behavioral skills and capacity:* Habit-building strategies may scaffold new behaviors gradually. |
| What are common psychological or environmental barriers that may prevent Malaysian YMSM from fully utilizing HIV prevention tools in the app, and how might the app design address these? | *Perceived and actual environmental constraints:* Directly targets barriers such as stigma, privacy concerns, connectivity, or language.  *Behavioral skills and capacity:* Touches on whether users feel equipped to navigate the app or understand the content. |
| What emotional drivers or personal values should the app appeal to increase motivation for HIV prevention behaviors? | *Affective motivation:* Explores emotional appeal, such as pride, love, belonging, or fear, that might drive protective behaviors. |
| What app features (e.g., storytelling, peer interaction) could make HIV prevention behaviors feel more meaningful and personally relevant for YMSM? | *Salience of behavior:* Personal stories and relatable content increase perceived importance of the behavior.  *Affective motivation:* Stories and peer encouragement can evoke emotional resonance.  *Behavioral skills and capacity:* Suggestions from peer users may adopt. |
| How can the *MY-Hero* app better support users in developing the skills needed to access, negotiate, or adhere to HIV prevention methods (e.g., PrEP, testing, condom use)? | *Behavioral skills and capacity:* Directly address teaching or reinforcing core skills like content library.  *Environmental constraints:* May uncover structural barriers to access HIV prevention services (e.g., where to get PrEP). |
