# Supplementary material for: Designing a Gamified mHealth App for HIV Prevention and Comorbidities Among Malaysian Young Men Who Have Sex With Men: An Interdisciplinary Expert Panel Study
Source: J Med Internet Res. 2026 Sep 8;28:e80334. doi: 10.2196/80334 (PMC13552997; doi:10.2196/80334)
Supplement: Supplementary file 3 [file jmir-v28-e80334-s003.docx]

**Appendix 3.** Operationalization, Trigger Mechanisms, and Targeted Behavioral Determinants of Gamification Elements in the MY-Hero App

| **Gamification Elements** | **Behavioral Target** | **Operationalization in MY-Hero** | **Trigger Mechanism** | **Reward/Feedback Type** | **User Data Tracked** | **IBM Construct Targeted** |
| --- | --- | --- | --- | --- | --- | --- |
| Achievement   | Encourage completion of prevention-related activities and engagement | Users complete goals for learning modules, quizzes, articles read, testing kits ordered, within the app | Completion of each goal | In-app virtual badges; haptic feedback; progress indicators | Module completion, quiz results, activity completion | Affective motivations; Salience of the behavior |
| Avatars   | Enhance personalization and user identification with the app environment | Users select and customize a Malaysian-style avatar representing their in-app identity | Avatar selection during onboarding and customization unlocked through competing goals | In-app customization options (appearance, accessories) | Avatar selection, customization choices | Affective motivations; Salience of the behavior |
| Collections   | Encourage repeated engagement and exploration of app | Users collect virtual rewards (i.e., badges) by completing tasks and goals | Completing modules, quizzes, or specific in-app challenges | Digitally collectible badges displayed in a collection page | Collected items, completed activities | Prior habits; Affective motivations |
| Rewards   | Reinforce desired health behaviors and app engagement | Users receive in-app points for redeeming actual items like T-shirt, power bank, etc | Completion of learning activities, quizzes, or regular engagement | Virtual points that accumulate in the user profile | Points earned, activity frequency | Affective motivations; Prior habits |
| Unlocking content   | Encourage progression through educational material and app usage | Additional avatar features like accessories, hair styles become available as users progress | Completion of prerequisite modules or reaching threshold points | Access to more avatar features | completed activities | Affective motivations; Salience of the behavior |
| Badges   | Recognize accomplishments and reinforce motivation | Users earn in-app visual badges for completing goals or maintaining engagement streaks | Achieving predefined activities | Digitally collectible badges displayed in a collection page | Badge attainment, goal completion | Affective motivations; Salience of the behavior |
| Feedback   | Reinforce learning and guide behavior change | Immediate haptic feedback is provided after quizzes or activities, including correct answers and health information | Completion of quizzes or learning activities | Congratulation messages and haptic feedback like a vibration | Quiz responses, knowledge scores | Behavioral skills and capacity; Salience of the behavior |
| Leaderboard   | Encourage friendly competition and social motivation | Users can see their ranking relative to other users based on in-app activity levels | Accumulated points | Ranking display comparing user progress with others | Points, in-app activities metrics | Affective motivations; Salience of the behavior |
| Surprising gifts   | Sustain engagement through unpredictability | Users occasionally receive random mailed surprise party pack | Randomized distribution | Unexpected surprise party pack that includes harm reduction supplies | Login frequency, activity completion | Affective motivations |
| Progression system   | Maintain long-term engagement through visible progress | A progress bar or level system reflects advancement through content and activities | Accumulated points and completed goals | Level advancement and visual progress indicators | Points, completed modules, goals, and quizzes | Prior habits, behavioral skills and capacity |
