# Supplementary material for: Designing a Gamified mHealth App for HIV Prevention and Comorbidities Among Malaysian Young Men Who Have Sex With Men: An Interdisciplinary Expert Panel Study
Source: J Med Internet Res. 2026 Sep 8;28:e80334. doi: 10.2196/80334 (PMC13552997; doi:10.2196/80334)
Supplement: Supplementary file 4 [file jmir-v28-e80334-s004.docx]

**Appendix 4.** Screenshots of the main features of the MY-Hero app.
